# Proinflammatory Action of a New Electronegative Low-Density Lipoprotein Epitope

**DOI:** 10.3390/biom9080386

**Published:** 2019-08-20

**Authors:** Tanize do Espirito Santo Faulin, Soraya Megumi Kazuma, Gustavo Luis Tripodi, Marcela Frota Cavalcante, Felipe Wakasuqui, Cristiano Luis Pinto Oliveira, Maximilia Frazão de Souza Degenhardt, Jussara Michaloski, Ricardo José Giordano, Daniel Francisco Jacon Ketelhuth, Dulcineia Saes Parra Abdalla

**Affiliations:** 1 Department of Clinical and Toxicological Analyses, Faculty of Pharmaceutical Sciences, University of Sao Paulo, Sao Paulo 05508-000, Brazil; 2 Department of Experimental Physics, Institute of Physics, University of Sao Paulo, Sao Paulo 05508-090, Brazil; 3 Department of Biochemistry, Institute of Chemistry, University of Sao Paulo, Sao Paulo 05508-000, SP, Brazil; 4 Centre for Molecular Medicine, Department of Medicine, Karolinska University Hospital, Karolinska Institute, 17164 Stockholm, Sweden; 5 Department of Cardiovascular and Renal Research, Institute for Molecular Medicine, University of Southern Denmark (SDU), 5000 Odense, Denmark

**Keywords:** atherosclerosis, macrophages, LDL, oxidized LDL, electronegative LDL, danger-associated molecular pattern, mimotope peptide, phage display, epitope mapping

## Abstract

The electronegative low-density lipoprotein, LDL (−), is an endogenously modified LDL subfraction with cytotoxic and proinflammatory actions on endothelial cells, monocytes, and macrophages contributing to the progression of atherosclerosis. In this study, epitopes of LDL (−) were mapped using a phage display library of peptides and monoclonal antibodies reactive to this modified lipoprotein. Two different peptide libraries (X6 and CX8C for 6- and 8-amino acid-long peptides, respectively) were used in the mapping. Among all tested peptides, two circular peptides, P1A3 and P2C7, were selected based on their high affinities for the monoclonal antibodies. Small-angle X-ray scattering analysis confirmed their structures as circular rings. P1A3 or P2C7 were quickly internalized by bone marrow-derived murine macrophages as shown by confocal microscopy. P2C7 increased the expression of TNFα, IL-1 β and iNOS as well as the secretion of TNFα, CCL2, and nitric oxide by murine macrophages, similar to the responses induced by LDL (−), although less intense. In contrast, P1A3 did not show pro-inflammatory effects. We identified a mimetic epitope associated with LDL (−), the P2C7 circular peptide, that activates macrophages. Our data suggest that this conformational epitope represents an important danger-associated molecular pattern of LDL (−) that triggers proinflammatory responses.

## 1. Introduction

The pathogenesis of atherosclerosis involves innate and adaptive immune responses. A high blood level of low-density lipoproteins (LDLs) is considered a major risk factor for atherosclerosis due to increased subendothelial retention of LDL particles, their modification, and the production of new autoantigens that trigger maladaptive immune responses in the arterial wall [1,2]. In this context, a subfraction of modified LDLs found in blood and characterized by high electronegativity [3,4,5] is called electronegative LDL [LDL (−)], and has been shown to trigger proinflammatory responses in endothelial cells and macrophages [6,7,8,9]. More recently, LDL (−) has been found to interact with CD14 and TLR4 in human monocytes and macrophages, which implicates LDL (−) as a potential important transducer of proinflammatory effects [10]. In addition to affecting innate immune responses, LDL (−) and its autoantibodies have been detected in blood plasma and atherosclerotic lesions of humans and rabbits [11,12]. Although different lipids derived from LDL have been identified as triggers of inflammatory responses, some studies have indicated that the LDL protein moiety can be bioactive. Several peptides derived from apoB-100 have been shown to activate T and B cells [13,14,15] and also demonstrated anti-inflammatory activity [16]. Another peptide, apoB-100 danger associated signal 1 (ApoBDS-1) has been shown to induce the release of IL-6, IL-8, and CCL2 by monocytes, macrophages, and atherosclerotic plaque cultures [14] as well as activate platelets [17]. In previous work, we described two monoclonal antibodies (mAbs), 1A3 and 2C7, capable of recognizing different epitopes of LDL (−). These mAbs were used to develop ELISA-based assays for the quantification of LDL (−) and its autoantibodies [18]. Using these assays, we showed that LDL (−) and its autoantibodies were increased in individuals with high atherosclerosis risk, such as chronic coronary syndromes [19]. Furthermore, passive immunization with these anti-LDL (−) antibodies has been shown to be atheroprotective in Ldlr ^−/−^ mice [20]. Following this work, other studies have produced an anti-LDL (−) single-chain fragment variable (scFv) of the 2C7 monoclonal antibody that has potential applications in atherosclerosis therapy and diagnosis [21,22,23]. However, the molecular properties of immunodominant proinflammatory epitopes of LDL (−) remain unknown. In this study we mapped epitopes of LDL (−) recognized by the 1A3 and 2C7 mAbs in a phage display library and investigated their capacity to stimulate macrophages in vitro. 

## 2. Materials and Methods 

### 2.1. Materials 

RPMI 1640, fetal bovine serum, streptomycin sulfate, penicillin G sodium and amphotericin B were purchased from Gibco BRL (Gaithersburg, MD, USA). Mineral oil (Nujol) was purchased from Schering-Plough (Sao Paulo, Brazil). Phenyl methyl sulfonyl fluoride (PMSF) was purchased from Sigma-Aldrich (St Louis, MO, USA). A Protein G column and a PD-10 desalting column were purchased from GE Healthcare (Uppsala, Sweden). All synthetic peptides, including FITC-labeled peptides, were synthesized and purified by HPLC to purities greater than 95% by the Chinese Peptides Company (Hangzhou, China). The 1A3H2 and 2C7D5F10 anti-LDL (−) mAbs were obtained as previously described [11].

### 2.2. Phage Display Random Peptide Libraries 

Two phage display random peptide libraries were previously constructed on vector fUSE55 displaying inserts X6 or CX8C (C indicates cysteine; X indicates any amino acid) that were fused to an outer minor phage coat protein (pIII) at the N-terminus of an Fd phage [24,25]. These tetracycline-resistant libraries consisted of a linear, 6-peptide configuration (X6), or a cyclic, 8-peptide configuration (CX8C) with a loop scaffold developed by flanking the random peptide sequence with a pair of cysteine residues to form a bridge. The details of biopanning, binding assays, and ELISA procedures with synthetic peptides are described in the Supplementary Materials (A. Phage Display).

### 2.3. Characterization of the Peptides by Small-Angle X-ray Scattering (SAXS)

SAXS experiments were performed with SAXS Xenocs-Xeuss^TM^ (Xenocs, Sassenage, France) with a GENIX source and Fox2D mirrors. Collimation was performed using two sets of scatterless slits that provide a high flux (approximately 0.5 × 10^8^ ph/s/mm^2^) with very low parasitic scattering. Data treatment was performed using the software package SUPERSAXS, and the final scattering intensity was normalized to an absolute scale based on water as the primary standard. The normalization of the data to an absolute scale permitted the estimation of the average molecular weight of the particles [26]. The methodological details are described in the Supplementary Materials (B) SAX Structure Characterization.

### 2.4. Isolation of Human LDL (−)

Venous blood from normocholesterolemic volunteers was collected into tubes containing ethylenediamine tetraacetic acid (EDTA, 1 mg/mL blood). Pooled plasma was immediately separated by centrifugation, at 2500 rpm for 10 min at 4 °C and 1 mM (PMSF); 2 mM benzamidine, 2 μg/mL aprotinine and 20 mM butylated hydroxytoluene (BHT) were added to prevent protease activity and oxidative reactions. LDL (d ~ 1.019 to 1.063 g/mL) was isolated from plasma by preparative sequential ultracentrifugation, using an Optima XE-90 ultracentrifuge (Beckman Coulter, IN, USA) with a fixed angle rotor (T 70.1). Plasma was centrifuged with saline (density = 1.019 g/mL) at 56,000 G for 7 h at 4 °C and the top fraction was removed. The remaining plasma was adjusted with KBr to a density of 1.063 g/mL and centrifuged with saline (density = 1.063 g/mL). The top LDL fraction was removed and dialyzed against 20 mM Tris-HCl buffer, pH 7.4, 20 mM BHT, and 10 mM EDTA for 4 h, at 4 °C, protected from light, with buffer exchanges each 1 h. The LDL (−) was separated from the native LDL (nLDL) by fast protein liquid chromatography (FPLC, ÄKTA Start, GE Healthcare, Chicago, Illinois, USA) using an ion exchange column (Sepharose UNO Q, Bio-Rad Laboratories Inc, Hercules, CA, USA). Total LDL was injected into the column pre-equilibrated with buffer A (10 mM Tris-HCl, pH 7.4) and eluted with a multi-step gradient of buffer B (10 mM Tris-HCl, 1 M NaCl). The isolated LDL (−) fraction was dialyzed against phosphate buffered saline (PBS) and concentrated using a specific device (Vivaspin 20, 100,000 MWCO, GE Healthcare Life Sciences, Uppsala, Sweden). The presence of LPS in LDL samples isolated from human plasma was determined by the limulus amebocyte lysate (LAL) test (Lonza, Verviers, Belgium). Supplies used for LDL isolation and cell culture were treated with E-Toxa-Clean ^®^Concentrate (Sigma Aldrich, St. Louis, MO, USA). The endotoxin levels of LDL (−), P1A3, and P2C7 peptides were < 0.1 EU (endotoxin unit) for all samples.

### 2.5. Obtention of Bone Marrow-Derived Macrophages

Bone marrow-derived macrophages (BMDMs) were obtained by extracting bone marrow cells from femurs of C57BL/6 mice according to [27]. Further, bone marrow cells were differentiated to macrophages through incubation with RPMI 1640 media, with 10 ng/mL of recombinant M-CSF (Peprotech, Rocky Hill, NJ, USA) and 15% fetal bovine serum at 37 °C, 5% CO_2_ in humidified air. BMDMs were characterized using flow cytometry and an F4/80-PE-Cy5 antibody (eBioscience, San Diego, CA, USA), which is a specific antibody marker for murine macrophages. Labeled cells were analyzed in a flow cytometer (FACS Canto, BD Biosciences, San Diego, CA, USA). FACS characterization of macrophages is shown in the Supplementary Materials (C. Macrophage characterization; Figure S6).

### 2.6. Peptide Endocytosis by Macrophages

After differentiation, BMDMs (10^5^ cells/well) were plated on glass-bottomed culture plates (35 mm diameter with 4 wells) (Greiner Bio-One, Kremsmunster, Austria). The cells were maintained in 1% FBS before treatment. Then, the cells were incubated with 100 μg/mL Cy5-P1A3 and Cy5-P2C7 for 3 and 6 h. Further, the cells were fixed in 4% paraformaldehyde before visualization under a Zeiss LSM 780-NLO confocal microscope (Carl Zeiss, Jena, Germany). In order to investigate if the internalization of peptides by macrophages occurred by endocytosis, inhibitors of this process were used as follows. For this assay, 10^6^ cells/well were incubated for 1 h with the following inhibitors: 1 mM sodium azide (oxidative phosphorylation inhibition), 1 μM Brefeldin A (Golgi complex/endoplasmic reticulum protein traffic inhibition), 40 μM Cytochalasin D (phagocytosis inhibition), 80 μM Dynasore (dynamin-dependent endocytosis inhibition), and 20 μM sodium monensin (lysosome enzyme inhibition). The cells were washed with PBS, then 100 μg/mL Cy5-P1A3 or Cy5-P2C7 were added and the cells were incubated for up to 2 h.

### 2.7. Macrophage Activation

BMDMs (10^5^ cells/well) were incubated with synthetic peptides and 100 μg/mL LDL (−) or PBS as negative control and 10 μg/mL LPS as positive control for 3 h at 37 °C. Total RNA was extracted with TRIzol (Life Technologies, Carlsbad, CA, USA) following the manufacturer’s instructions. The extracted RNA was quantified by spectrophotometry. cDNA was constructed from RNA samples by RT-PCR with Superscript Vilo (Life Technologies, Carlsbad, CA, USA). qPCR was performed using the 7500 Fast Real-Time PCR System (Applied Biosystems, Carlsbad, CA, USA) to analyze the expression levels of NOS2, COX-2, TNF-α, IL-1α, TGF-β, and IL-10. Activation of macrophages by LPS was evaluated as a positive control (Supplementary Materials (C. Macrophage characterization; Figure S7)). To verify if BMDM activation was dependent on the endocytosis of peptides an experiment was carried out with Brefeldin A. For this 10^6^ BMDM/well were treated for 1 h with 1 μM Brefeldin A. Then, the cells were washed with PBS and incubated with 100 μg/mL P2C7 in RPMI 1640, 10% FBS and Brefeldin A (1 μM) for 3 h. A negative control was done only with RPMI 1640, 10% FBS and Brefeldin A (1 μM) incubated for 3 h. Further the expression of NOS2, TNF-α and TGF-β was evaluated by qPCR assay.

### 2.8. Evaluation of BMDM Polarization by Flow Cytometry

For this assay, 10^6^ cells/well were incubated for 48 h with 100 μg/mL P2C7 or PBS as the negative control. After incubation, macrophages were washed with PBS and removed from the plate with cold PBS (plus 10 mM EDTA). The cells were stained with anti CD206—FITC (Invitrogen, MR5D3), anti-F4/80—PE-Cy5 (eBioscience, BM8), anti-CD80-PE (eBioscience, 16-10A1), anti-F4/80—FITC (BM8), anti-MHC II—APC-Cy7 (Invitrogen, M5/114.15.2) and anti-CD86—APC (Invitrogen, GL1) in PBS, 4% FBS for 30 min. Further labeled cells were analyzed in a flow cytometer (FACS Canto, BD Biosciences, San Diego, CA, USA). FACS characterization of M2 macrophages is shown in the Supplementary Materials (C. Macrophage characterization; Figure S8).

### 2.9. Cytokine Quantification by Cytometric Bead Array (CBA) Kit

BMDMs (10^6^ cells/well) were incubated with synthetic peptides at 100 μg/mL for 24 h in an incubator at 37 °C. Then, the medium was collected and stored at −80 °C. Cytometric bead array (CBA) mouse inflammation kits (BD Biosciences, San Diego, CA, USA) were used according to manufacturer instructions to quantify the levels of IL-12p70, TNF-α, INF-γ, CCL2, IL-10, and IL-6 cytokines. 

### 2.10. Nitric Oxide Quantification (NOA)

The NO concentration in the culture medium was evaluated by measuring the NO^2−^ accumulation with a nitric oxide analyzer (NOA^TM^ 280; Sievers Inc., Boulder, CO, USA) that is based on a gas-phase chemiluminescence reaction between NO and ozone. In brief, BMDMs (2 × 10^6^ cells) were treated for 24 h with 100 μg/mL LDL (−), P1A3 or P2C7, and 100 μL of culture supernatants were injected into a reflux chamber containing vanadium (III) in 3N HCl heated to 90 °C. The NO produced was detected by gas phase chemiluminescence after reaction with ozone. A calibration curve was then created using a sodium nitrate standard solution.

### 2.11. Statistical Analysis

Statistical analyses were performed using GraphPad Prism software (version 6.0, San Diego, CA, USA) and one-way ANOVA followed by Tukey–Kramer or Dunnett’s tests for multiple comparisons as well as t-tests for comparisons between data pairs.

### 2.12. Data Availability

The data sets that were generated and/or analyzed during the current study are available from the corresponding author upon reasonable request.

### 2.13. Human and Animal Rights

The study was approved by the Committee on Ethics in Human Research (CEP) for the use of human blood (protocol number 557), and by the Committee on Ethics in Animal research (CEUA) for the use of C57BL/6J (protocol number 492), both of the Faculty of Pharmaceutical Sciences from the University of Sao Paulo, in agreement with the National Committee on Ethics in Research (CONEP) and the Brazilian College for Animal Experimentation (CONCEA) guidelines. Informed consent was obtained from all study subjects.

## 3. Results

### 3.1. Mapping of LDL(-) Mimetic Epitopes

**Figure 1:**
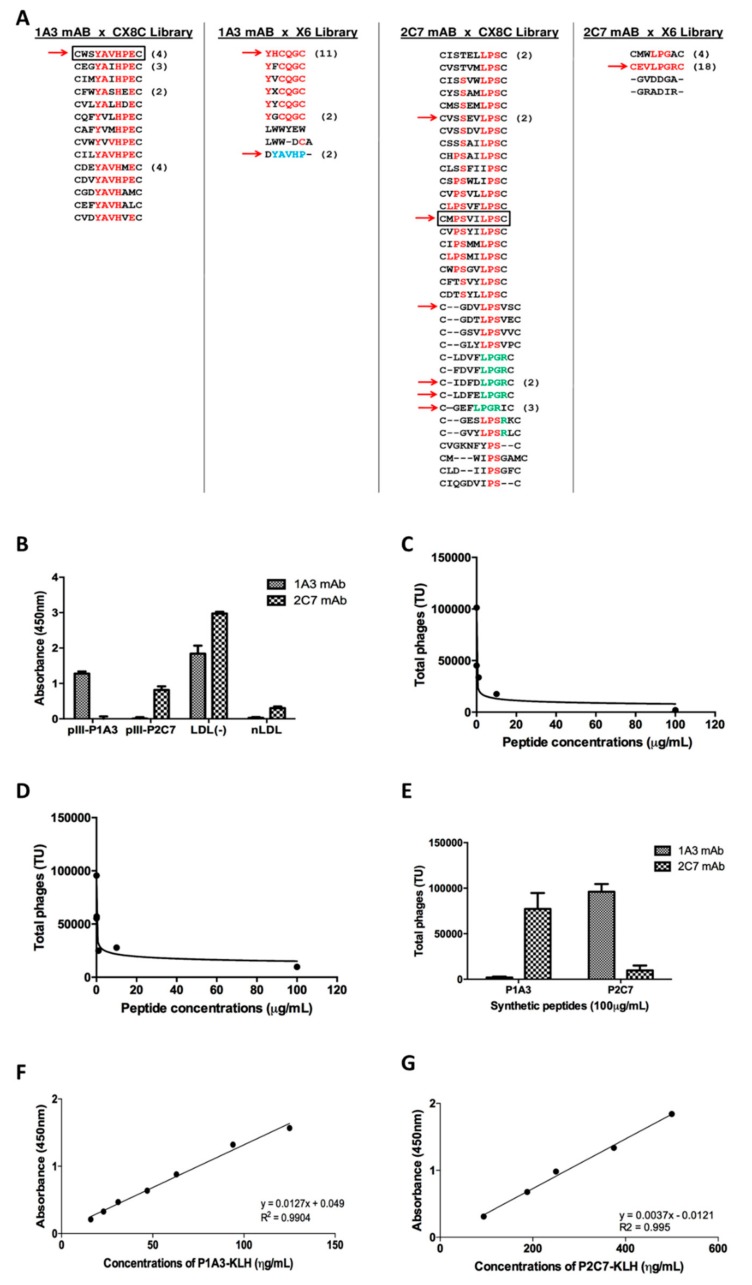
Anti LDL (−) monoclonal antibody ligands. (**A**): The peptides identified in each biopanning were aligned using Clustal Omega. Common amino acids are highlighted in bold and blue. Red indicates hydrophobic residues. All the epitopes were obtained after four rounds of panning, except epitopes from 2C7 mAb with X6 library, which were obtained after three selection rounds. The frequency of identical peptides is indicated in parentheses. The red arrows indicate the peptides that have been chosen to be tested in a binding assay with their respective mABs. Regarding the mAB 1A3, the blue peptides selected from the X6 library have the same amino acid sequence as the peptides selected by the CX8C library. Regarding mAB 2C7, the green peptides selected from the CX8C library have the same amino acid sequence (LPGR) of the peptides selected by the X6 library. The peptides highlighted in a box were used for subsequent assays. (**B**): The immunoassay demonstrated the affinity of 1A3 mAb for pIII-P1A3 and the affinity of 2C7 mAb for pIII-P2C7 (100 μg/mL coated on the plate) using LDL (−) as a positive control and native LDL as a negative control. (**C**,**D**). Competition binding for the 1A3 (**C**) and 2C7 (**D**) mAbs coated on plates with phages presenting P1A3/P2C7 peptides that were incubated in increasing concentrations of correspondent synthetic peptide on the plate. (**E**–**G**): Inhibition of phage binding was found for the cognate peptide. The P1A3 peptide did not inhibit the binding of phage 2 for 2C7 mAb, and the P2C7 peptide did not inhibit the binding of phage 1 for 1A3 mAb (**E**). F,G: ELISA assay with the KLH-derivatized synthetic peptides. The immunoassay demonstrates the affinity of 1A3 mAb for KLH-coated P1A3 (**F**) and the affinity of 2C7 mAb for KLH-coated P2C7 (**G**). The bars indicate the mean ± SD of triplicates.

To prevent the selection of peptides binding to conserved domains of immunoglobulins, the anti-LDL (−) mAbs 1A3 and 2C7 were immobilized on microtiter plates and incubated in the presence of excess soluble, unrelated mouse immunoglobulin. After three selection rounds, we observed significantly enriched phage binding (Figures S1 and S2). After sequencing to identify the peptides displayed by individual phage particles, the biopanning results showed specific enriched peptide sequences because of the repeating motifs in the isolated peptide sequences (Figure 1A).

For binding assays, phages CWSYAVHPEC, YHCQGC, and DYAVHP were selected from the biopannings of mAb 1A3, and phages CEVLPGRC, CMPSVILPSC, CLDFELPGRC, CIDFDLPGRC, CGEFLPGRIC, CGDVLPSVSC, and CVSSEVLPSC were selected from the biopannings of mAb 2C7. From all of the selected phages, only phages CWSYAVHPEC and YHCQGC showed high-affinity binding for mAb 1A3, and phages CMPSVILPSC and CLDFELPGRC showed a higher affinity for mAb 2C7 (Figure S3). These phages were selected for further experiments. No high-affinity peptides isolated from the X6 library were validated, which suggested that they were too small to fit the mAb binding pockets.

Recombinant peptides fused to domain-1 of the phage pIII coat protein were produced to confirm the specific binding of the 1A3 and 2C7 mAbs to their respective peptides by Western blot and ELISA (Figures S4 and S5 and Figure 1B). Competition-binding assays using pIII-D1-CWSYAVHPEC and pIII-D1-CMPSVILPSC phages as well as their respective synthetic peptides (P1A3 for CWSYAVHPEC and P2C7 for CMPSVILPSC) showed that increased concentrations of the synthetic peptides inhibited the binding of each phage to the 1A3 or 2C7 mAbs (Figure 1C–E), which confirmed their specificities. Additionally, ELISAs with the KLH-derivatized synthetic peptides also indicated specific binding to the 1A3 and 2C7 mAbs (Figure 1F,G). These two synthetic peptides (CWSYAVHPEC and CMPSVILPSC, which were named P1A3 and P2C7) were subsequently tested for biological activity.

### 3.2. Structural Characterization of P1A3 and P2C7

**Figure 2:**
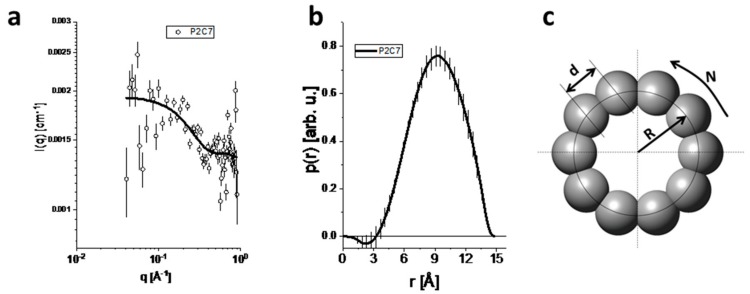
Experimental SAXS data for sample P2C7 and indirect Fourier transform (IFT) modeling. (**a**) SAXS data obtained at a distance of 38 cm between the sample and the detector (symbols) together with the IFT fit (solid line). (**b**) p(r) curve calculated by the IFT procedure. (**c**) Sketch of the circular ring construction from SAXS modeling data; N: number of amino acids; R: radius; d: distance between the radii of two amino acids.

To confirm the cyclic structure of the peptides, a SAXS study was performed. P1A3 and P2C7 peptides are expected to form circular rings with ten amino acids and an approximate molecular weight (MW) of 1.5 kDa. The theoretical sizes of such rings, which are assumed to have a typical distance of 3.6 Å between amino acids, would be 12–14 Å in diameter or to have an approximately 6–7 Å radius. The theoretical intensity of this circular ring ranged from 0.05 Å^−1^ ≤ q ≤ 1.0 Å^−1^. Within this range, information about the ring structure can be determined. The SAXS data and obtained p(r) curve for P2C7 are shown in Figure 2a,b. Interestingly, the shape of the p(r) curve is similar to the curves obtained for hollow objects [27], which was expected for circular rings. Further results are shown in Tables S1 and S2 (Supplementary Materials) for both peptides. The D_max_ value obtained for the data at 38 cm was similar to the theoretical size of the circular ring (~14Å), which provides an important indication about the formation of peptide rings in the system. The estimated molecular weight of the scattering particle was 1.54 ± 0.16 kDa, which is consistent with the expected value for a 10-amino acid ring (Figure 2c). In summary, the SAXS results confirmed the cyclic structures of both peptides.

### 3.3. P1A3 and P2C7 Are Internalized by Macrophages

**Figure 3:**
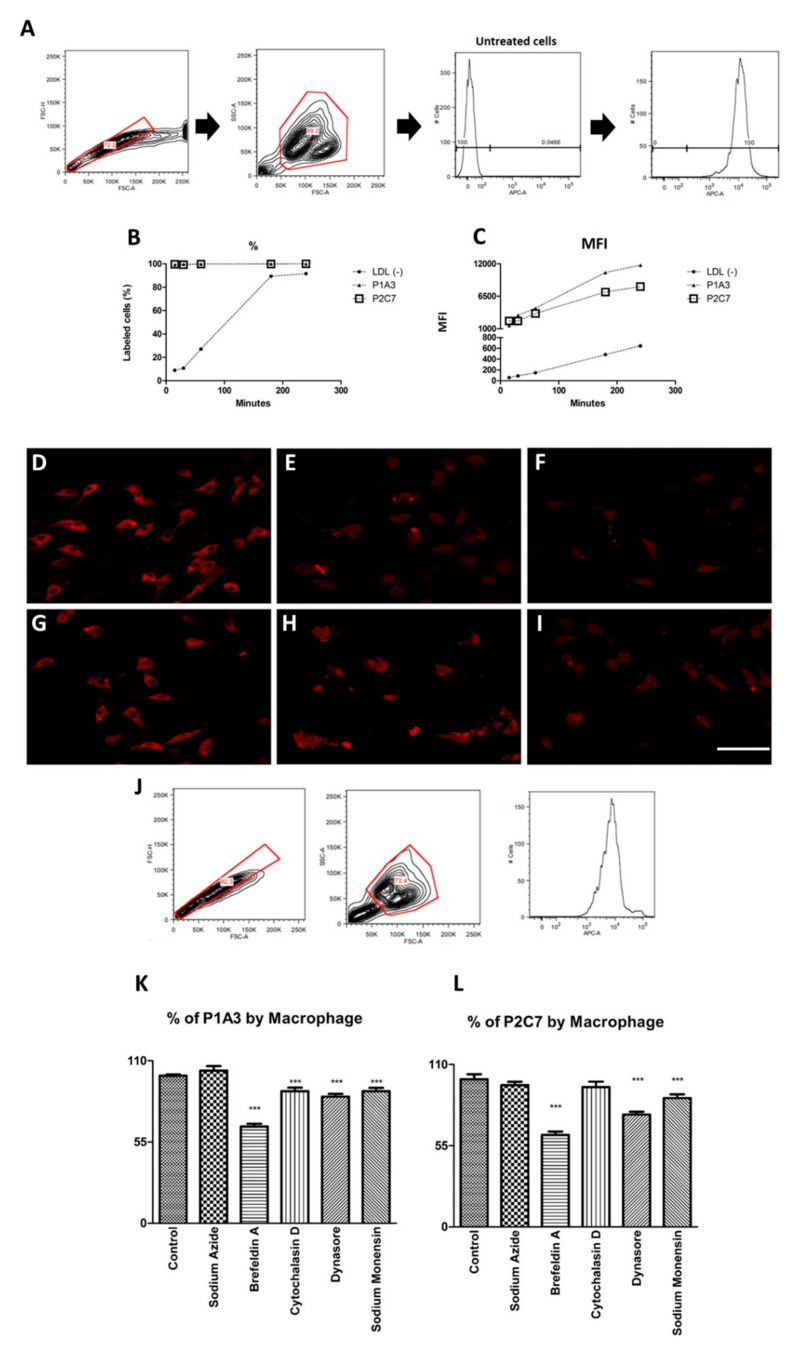
P1A3 and P2C7 internalization by macrophages. (**A**–**C**): Bone marrow-derived macrophages were incubated with 100 μg/mL of Cy5-P1A3, Cy5-P2C7, and Dil-LDL (−) and the cells were analysed by flow cytometry (**A**) at 15, 30, 60, 180, and 240 min. The percentage of labeled cells was demonstrated at these five timepoints (**B**). The median fluorescence intensity (MFI) from Cy5-P1A3, Cy5-P2C7, and Dil-LDL (−) was measured in macrophages (**C**). (**D**–**I**): 10^5^ cells were incubated with 100 μg/mL Dil-LDL (−) for 3 h (**D**) or 5 h (G); 100 μg/mL Cy5-P1A3 for 3 h (**E**) or 5 h (**H**); and 100 μg/mL Cy5-P2C7 for 3 h (**F**) or 5 h (**I**). The images were acquired using confocal microscopy. The white bar (**I**) represents 32 µm and the picture was taken with 5.1 μm of profundity. (**J**–**L**): 1 × 10^5^ bone marrow-derived macrophages (BMDMs) were treated with various endocytosis inhibitors for 1h before 3 h incubation with 100 μg/mL Cy5-P1A3 (**K**) or Cy5-P2C7 (**L**). The reading was done using flow cytometry and the median intensity fluorescence (MFI) was assessed to compare treated cells to untreated cells. The graph is shown as percentage of Cy5-P1A3/P2C7 according to cell amount. Statistical analyses were performed with one-way ANOVA followed by Dunnett’s test, *** *p* < 0.005; baseline treatment vs. inhibitor.

The binding of Cy5-labeled P1A3 and P2C7 peptides, or Dil-labeled LDL (−) in BMDMs is shown in Figure 3. After 15 min of incubation, 100% of the macrophages were labeled with Cy5-P1A3 and Cy5-P2C7, whereas the Dil-LDL(-) binding was slower (Figure 3B). The median fluorescence intensity (MFI) for both peptides increased over time, with a slightly higher slope for P1A3 compared to P2C7 and LDL (−) (Figure 3C). Confocal microscopy (Figure 3D–I) indicated that both Cy5-labeled peptides were internalized by BMDMs and appeared as small intracellular droplets, whereas Dil-LDL (−) were spread throughout the cytoplasm. P1A3 endocytosis by BMDMs was inhibited by Brefeldin A, cytochalasin D, dynasore, and sodium monensin, whereas P2C7 endocytosis was inhibited by Brefeldin A, dynasore, and sodium monensin (Figure 3J–L). For both peptides, Brefeldin A provided the most significant endocytosis inhibition.

### 3.4. P2C7 Is a Proinflammatory Stimulus to Macrophages

**Figure 4:**
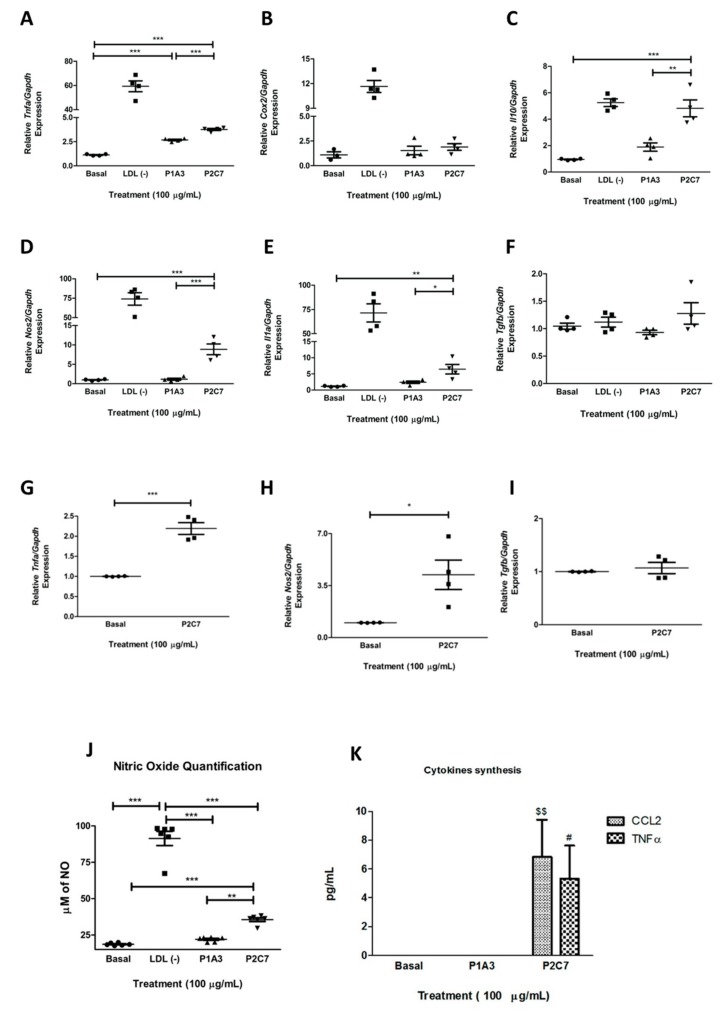
Effects of P1A3 and P2C7 treatment on macrophages. (**A**–**F**): Proinflammatory (TNF-α, COX-2, NOS2, and IL-1α; (**A**, **B**, **D**, and **E**, respectively) and anti-inflammatory (TGF-β and IL-10; C and F, respectively) cytokine gene expression was evaluated by qPCR. The results are expressed as mean ± SD; *** *p* < 0.005, * *p* < 0.05. The comparisons P1A3 vs. P2C7 vs. basal were undertaken with one-way ANOVA followed by the Tukey test. (**G**–**I**): Proinflammatory (TNF-α and NOS2; G and H, respectively) and anti-inflammatory (TGF-β; I) cytokine gene expression was evaluated after treatment of BMDM with p2C7 in the presence of Brefeldin A. The results are expressed as mean ± SD, *** *p* < 0.005, ** *p* < 0.001 and * *p* < 0.05. The comparison P2C7 vs. basal was done by Student’s t-test. (**J**): Nitric oxide synthesis was measured by ozone-chemiluminescence technology with a nitric oxide analyzer. (**K**): IL-12p70, TNF-α, IFN-γ, CCL2, IL-6, and IL-10 were analyzed with a cytometric bead array (CBA) mouse inflammation kit in the supernatant of BMDM treated with 100 μg/mL of mimotope peptides and at basal condition. IL-12p70, IL-6, IFN-γ or IL-10 concentrations were below the kit detection limit. The results are expressed as mean ± SD. *** *p* < 0.005, $ *p* < 0.01 comparing CCL2 synthesis and # *p* < 0.05 comparing TNF-α production. The statistical analyses were undertaken with one-way ANOVA followed by the Tukey test.

The effects of the P1A3 and P2C7 peptides on the expression of proinflammatory mediators in BMDMs are shown in Figure 4. LDL (−) induced a significant proinflammatory effect by increasing the mRNA expression of TNF-α, IL-1 α, COX-2, and NOS2. IL-10 is also induced by LDL (−) as previously reported (5). Only P2C7 stimulated the expression of TNF-α, IL-1 α, NOS2, and IL-10, which demonstrates that this peptide mimics the action of LDL (−) on macrophages although at less intensity (Figure 4A–F). In addition, macrophage activation by P2C7 does not depend on its internalization considering that Brefeldin A did not inhibit the expression of TNF-α and NOS2, as shown in Figure 4G–I. Moreover, both LDL (−) and P2C7 increased NO production compared to the control and the P1A3 peptide (Figure 4J). Furthermore, only P2C7 increased CCL2 and TNF-α secretion compared to P1A3 and control (Figure 4K).

**Figure 5:**
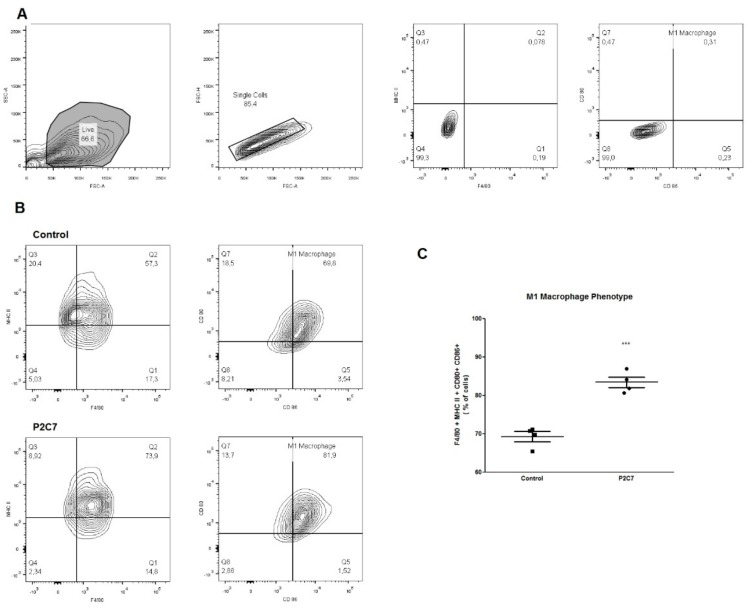
The P2C7 treatment induces M1 phenotype in BMDM. 1 × 10^6^ BMDMs were treated with 100 µg/mL P2C7 or not treated (control) for 48 h. After incubation, the cells were washed and stained with anti-F4/80-FITC, anti-MHC II-APC-Cy7, anti-CD80-PE, and and-CD86-APC. After selecting the gates with non-stained cells (**A**), the BMDMs treated with P2C7 promoted an increase of the M1 macrophages population (**B**,**C**). The statistical analyses were performed with Student’s t-test, *** *p* < 0.005 (*n* = 4).

To reinforce the evidence of the BMDM phenotype under P2C7 stimulation, the following markers were analyzed: M1 phenotype (MHC II, CD80, and CD 86), and M2 phenotype (CD206). P2C7-treated macrophages increased the M1 phenotype population (Figure 5) without affecting the M2 population (Figure S8).

## 4. Discussion

In vivo modified LDL particles, i.e., LDL (−), have proinflammatory characteristics that contribute to atherosclerosis progression [3,9]. Two peptides (P1A3 and P2C7) recognized by anti-LDL (−) monoclonal antibodies were identified in a phage display library. Although the two different X6 and CX8C libraries were used for peptide selection, the same motifs were found for the 1A3 (YAVHP) and 2C7 (VLPS) mAbs in both libraries. It is noteworthy that neither of the motifs resemble any part of the amino acid linear sequence of apoB-100 that accounts for 95% of the protein portion of LDL [28]. The structure recognized by the monoclonal antibodies is composed of segments of the protein that have discontinuities in the antigen amino acid sequence but are brought together in the three-dimensional structure of apoB-100, which strongly suggests that the P1A3 and P2C7 peptides correspond to conformational epitopes of LDL (−). Although our hypothesis is that both peptides are microdomains of the LDL (−) major protein (ApoB-100), we cannot exclude the possibility that these peptides may also be associated with some LDL (−) minor protein component previously described (28). These LDL (−) epitopes were probably exposed only when native LDL underwent modifications from oxidation, proteolysis, and glycation, among others, which possibly gave rise to conformational changes, since the mimotopes do not correspond to the linear sequence of ApoB-100. Additionally, small-angle X-ray scattering (SAXS) analyses of the peptides confirmed a cyclic structure with ring diameters of approximately 12–14 Å and approximate molecular weights of 1.54 kDa.

Interactions with macrophages showed that the binding and internalization of Cy5-labeled P1A3 peptide was higher compared to Cy5-P2C7. Both peptides were internalized by macrophages more rapidly than LDL (−) particles. The internalization of both peptides was primarily inhibited (40%) by Brefeldin A, which interrupts protein trafficking through the Golgi/endoplasmic reticulum complex and the recycling of endocytosed vesicles and integrins [29]. Dynasore also inhibited endocytosis, indicating that dynamin-dependent uptake also contributes to macrophage internalization of peptides.

Macrophages are vital cells in innate immunity and are a source of anti- and proinflammatory cytokines. Classical activators of macrophages include PAMP, LPS, or IFN-γ, but these cells can also respond to endogenous damage signals and stress. In response to DAMP (damage-associated molecular patterns), macrophages undergo physiological changes that lead to the production of proinflammatory cytokines and high levels of nitric oxide (NO) in a process known as polarization [30]. The polarization of macrophages has been widely studied and its role has gained prominence in several pathologies ranging from cancer to cardiovascular diseases [30]. Macrophage phenotypes can be grouped into two large families according to their characteristics and activation, including M1 macrophages (classic activation) and M2 macrophages (alternative activation) [30]. It has been shown in [31] that the presence of macrophages with these phenotypes in atherosclerotic plaques with M1 are considered to be atherogenic and have proinflammatory properties, while M2 appears to have a role in remodeling and healing, along with anti-inflammatory activity. Biochemically, the M1 macrophage mainly differs from M2 in arginine metabolism with the L-arginine/arginase pathway being primarily active, and the metabolism of l-arginine by NOS2 occurring only when it is stimulated [30].

The increase in P2C7-induced NOS2 expression suggests that this peptide may be a candidate for DAMP that can participate in the induction of the proinflammatory response of LDL (−) in macrophages, which could contribute to an increase in atherosclerotic plaque instability. Macrophage polarization for M1 was also confirmed by high NO (the final product of NOS2) release induced by P2C7, indicating a shift to the L-arginine/NOS2/NO pathway. These observations also strongly indicate that the P2C7 peptide mimics the macrophage activation effect of LDL (−). The induction of NOS2 by P2C7 peptide is also very relevant in the context of atherosclerosis. The upregulation of NOS2 expression in inflammatory states is related to the presence of pathogen molecules or cytokines that are highly associated with atherosclerosis. The inducible form of NOS releases high levels of NO that exacerbate inflammation, cellular damage, and apoptosis, which enhance atherogenesis [32,33]. NO is a potent mediator of homeostasis of the vascular endothelium. However, when provided by macrophages in the local oxidative stress environments of atherosclerotic lesions, NO is highly consumed by several reactions including that with O^−2^ (superoxide) to generate ONOO^−^ (peroxynitrite) that can oxidize LDL [34,35]. At high concentrations, peroxynitrite induces cytotoxic effects through peroxynitrous acid formation [36]. NOS2 expression in atherosclerotic lesions (humans/rabbits) has also been observed to co-localize with oxidation-specific epitopes (OSEs) from oxLDL [37]. Our data indicated that the P2C7 peptide induced a M1 macrophage response in addition to inducing the NOS2/NO pathway as well as the upregulation of TNF-α and IL-1α expression. This effect probably occurred via STAT1 activation with the consequent production of NO, and secretion of proinflammatory cytokines such as IL-1α and TNF-α. The IL-1α is constitutively expressed in several cells, even in healthy tissue [38]. However, the inducible expression of IL-1α can be upregulated by NF-κB and AP-1 transcription factors in response to TLR4 activators [39] or oxidative stress [40] and could be related to the overproduction of NO in P2C7-stimulated macrophages.

A myriad of inflammatory cytokines are released when macrophages are activated, which produces a microenvironment responsible for the proinflammatory state and leukocyte recruitment. The treatment of macrophages with P2C7 not only modulated the expression of genes related to M1 polarization but also increased the translation of proteins important for the proinflammatory response as TNF-α and CCL2. TNF-α is closely related to the modulation of the M1 phenotype and CCL2 is important in the recruitment of monocytes, memory T cells, and dendritic cells to sites of tissue injury. The expression of this proinflammatory chemokines is increased in atherosclerotic lesions [41], and CCL2-induced expression by oxLDL is associated with TNF-α [42]. Although TGF-β mRNA—a M2 polarization marker—was not upregulated by P2C7, the expression of IL-10 mRNA —an anti-inflammatory interleukin associated with the M2 phenotype—was induced by the P2C7 peptide and LDL (−). These observations agree with previous data showing the release of IL-10 from leukocytes treated with LDL (−) [33]. M2 macrophages can polarize into M2a (inflammation resolution/anti-inflammatory) and M2b/c (regulatory inflammation/anti-inflammatory [33]. In particular, M2b macrophage polarization occurs via proinflammatory stimuli, such as LPS or DAMPs plus IL-1β, and these cells express high levels of IL-10 along with inflammatory cytokines [43]. In this context, we can suggest that only a small population of BMDM was polarized to M2b by P2C7 increasing IL-10 mRNA without the corresponding increase of the cytokine in the supernatant of macrophage cultures, which probably occurred because its level was below the detection limit of the analytical method used in this study.

Innate and adaptive immune responses participate in all stages of atherosclerosis development. An innate immune response can be triggered by molecules derived from pathogens or self-antigens, i.e., pathogen-associated molecular patterns (PAMPs) and DAMPs, respectively. DAMPs are typically molecules that are internalized and released by immune cells during an injury, or simply molecules that changed into autoantigens. PAMPs and DAMPs are recognized by pattern recognition receptors (PRRs) which induce downstream signaling pathways that result in NFκB activation and proinflammatory gene expression [44]. The presence of PAMPs and DAMPs stimulates persistent innate immunological reactions in atherosclerosis progression. Among the identified atherosclerosis-related DAMPs, OSEs have been associated with multiple immunogenic effects [45,46]. Due to molecular mimicry, the immune response occurring in atherosclerosis can be the same for similar antigens. Therefore, because P2C7 is an antigenic mimetic part associated with LDL (−) and interacts with macrophages, this peptide may activate these cells upon recognition by PRRs to increase inflammatory responses in atherosclerotic plaques. Other studies have investigated native peptide fragments of apo-B100 [14], aldehyde-modified peptide sequences in apo-B100 [13], or peptide mimotopes of malondialdehyde-LDL [47]. However, in the present study we identified two LDL (−)-mimetic epitopes for the first time and described their effects on macrophages. The P2C7 peptide showed proatherogenic-related actions through stimulation of the expression of inflammatory mediators and the secretion of cytokines. Importantly, the P2C7 peptide induced inflammation by upregulating TNF-α, IL-1α, and NOS2 expression in macrophages. The polarization of macrophages in the atherosclerotic plaque is a very complex process owing to the several different stimuli that are produced by the microenvironment [48,49]. Considering the action of p2C7, it is possible that p2C7 peptide may be a potential antigen for active immunization in atherosclerosis prevention. The feasibility of a peptide-based therapy for atherosclerosis was investigated in previous studies [16,47], and a significant reduction of atherosclerosis in mice immunized with peptide epitopes of Apo B and malondialdehyde epitopes has been shown. Future immunization studies with LDL (−) mimetic peptides may be a path for developing an atheroprotective vaccine. In addition, the use of these peptides as imaging biomarkers is another approach that should be highlighted. The formation of macrophages loaded with modified LDL (foam cells) is a crucial step in the atherogenic process. Thus, the accumulation of these peptides in macrophages could be useful in PET/CT scans to investigate atherosclerotic lesions by using these tagged peptides as imaging agents, which can be explored in the future.

## 5. Conclusions

Although we do not provide information about the capacity of this peptide to induce the M1 macrophage phenotype in the atherosclerotic plaque, we hypothesize that the presence of p2C7 mimotope in the LDL (−) particle can be one of the triggers of this inflammatory cascade, contributing to the atherosclerotic process. Thus, P2C7 is a peptide that mimics some of the proinflammatory actions of LDL (−) particles on macrophages and may represent an important DAMP in atherosclerosis. 

## Supplementary Materials

The following are available online at https://www.mdpi.com/2218-273X/9/8/386/s1. Figure S1. Selection cycles of affinity using phages displayed by X6 and CX8C libraries with 1A3 mAb; Figure S2. Selection cycles of the phages with X6 and CX8C libraries with 2C7 mAb; Figure S3. Binding of selected phages to 1A3 (A) and 2C7 (B) mAb immobilized on 96-well plate; Figure S4. SDS-PAGE electrophoresis of peptide-pIII fusion after purification; Figure S5. Western blot analysis with peptides-pIII; Figure S6. Macrophage differentiation; Figure S7. Effect of LPS on mRNA expression in bone marrow-derived macrophages evaluated with qPCR; Figure S8. M2 macrophage phenotype. Table S1. Parameters obtained from the IFT fitting; Table S2. Parameters obtained from the model fitting assuming circular ring; Table S3. Primers sequence.

## References

[1] Weber C., Noels H. (2011). Atherosclerosis: Current pathogenesis and therapeutic options. Nat. Med..

[2] Eldika N. (2004). Atherosclerosis as an inflammatory disease: Implications for therapy. Front. Biosci..

[3] Avogaro P., Bon G.B., Cazzolato G. (1988). Presence of a modified low density lipoprotein in humans. Arteriosclerosis.

[4] Mello A.P.Q., Da Silva I.T., Abdalla D.S.P., Damasceno N.R.T. (2011). Electronegative low-density lipoprotein: Origin and impact on health and disease. Atherosclerosis.

[5] Estruch M., Sánchez-Quesada J.L., Ordóñez Llanos J., Benítez S. (2013). Electronegative LDL: A Circulating Modified LDL with a Role in Inflammation. Mediat. Inflamm..

[6] De Castellarnau C., Sánchez-Quesada J.L., Benítez S., Rosa R., Caveda L., Vila L., Ordóñez-Llanos J. (2000). Electronegative LDL from normolipemic subjects induces IL-8 and monocyte chemotactic protein secretion by human endothelial cells. Arterioscler. Thromb. Vasc. Biol..

[7] Abe Y., Fornage M., Yang C.-Y., Bui-Thanh N.-A., Wise V., Chen H.-H., Rangaraj G., Ballantyne C.M. (2007). L5, the most electronegative subfraction of plasma LDL, induces endothelial vascular cell adhesion molecule 1 and CXC chemokines, which mediate mononuclear leukocyte adhesion. Atherosclerosis.

[8] Faulin T.D.E.S., Cavalcante M.F., Abdalla D.S.P. (2010). Role of electronegative LDL and its associated antibodies in the pathogenesis of atherosclerosis. Clin. Lipidol..

[9] Pedrosa A.M.C., Faine L.A., Grosso D.M., De Las Heras B., Boscá L., Abdalla D.S.P. (2010). Electronegative LDL induction of apoptosis in macrophages: Involvement of Nrf2. Biochim. Biophys. Acta Mol. Cell Biol. Lipids.

[10] Estruch M., Bancells C., Beloki L., Sanchez-Quesada J.L., Ordóñez-Llanos J., Benitez S. (2013). CD14 and TLR4 mediate cytokine release promoted by electronegative LDL in monocytes. Atherosclerosis.

[11] Damasceno N.R.T., Sevanian A., Apolinário E., Oliveira J.M., Fernandes I., Abdalla D.S.P. (2006). Detection of electronegative low-density lipoprotein (LDL^−^) in plasma and atherosclerotic lesions by monoclonal antibody-based immunoassays. Clin. Biochem..

[12] Damasceno N.R.T., Apolinário E., Flauzino F.D., Fernandes I., Abdalla D.S.P. (2007). Soy isoflavones reduce electronegative low-density lipoprotein (LDL^−^) and anti-LDL^−^ autoantibodies in experimental atherosclerosis. Eur. J. Nutr..

[13] Fredrikson G.N., Hedblad B., Berglund G., Alm R., Ares M., Cercek B., Chyu K.-Y., Shah P.K., Nilsson J. (2003). Identification of Immune Responses Against Aldehyde-Modified Peptide Sequences in ApoB Associated with Cardiovascular Disease. Arter. Thromb. Vasc. Boil..

[14] Ketelhuth D.F.J., Rios F.J.O., Wang Y., Liu H., Johansson M.E., Fredrikson G.N., Hedin U., Gidlund M., Nilsson J., Hansson G.K. (2011). Identification of a Danger-Associated Peptide from Apolipoprotein B100 (ApoBDS-1) That Triggers Innate Proatherogenic Responses. Circulation.

[15] Hermansson A., Johansson D.K., Ketelhuth D.F., Andersson J., Zhou X., Hansson G.K. (2011). Immunotherapy with Tolerogenic Apolipoprotein B-100–Loaded Dendritic Cells Attenuates Atherosclerosis in Hypercholesterolemic Mice. Circulation.

[16] Kimura T., Kobiyama K., Winkels H., Tse K., Miller J., Vassallo M., Wolf D., Ryden C., Orecchioni M., Dileepan T. (2018). Regulatory CD4^+^ T Cells Recognize Major Histocompatibility Complex Class II Molecule—Restricted Peptide Epitopes of Apolipoprotein, B. Circulation.

[17] Assinger A., Wang Y., Butler L.M., Hansson G.K., Yan Z.Q., Söderberg-Nauclér C., Ketelhuth D.F.J. (2014). Apolipoprotein B100 danger-associated signal 1 (ApoBDS-1) triggers platelet activation and boosts platelet-leukocyte proinflammatory responses. Thromb. Haemost..

[18] Faulin T.D.E.S., De Sena K.C.M., Telles A.E.R., De Mattos Grosso D., Faulin E.J.B., Abdalla D.S.P. (2008). Validation of a novel ELISA for measurement of electronegative low-density lipoprotein. Clin. Chem. Lab. Med..

[19] Oliveira J.A., Sevanian A., Rodrigues R.J., Apolinário E., Abdalla D.S.P. (2006). Minimally modified electronegative LDL and its autoantibodies in acute and chronic coronary syndromes. Clin. Biochem..

[20] Grosso D.M., Ferderbar S., Wanschel A.C.B.A., Krieger M.H., Higushi M.L., Abdalla D.S.P. (2008). Antibodies against electronegative LDL inhibit atherosclerosis in LDLr-/-mice. Braz. J. Med Biol. Res..

[21] Kazuma S.M., Cavalcante M.F., Telles A.E., Maranhão A.Q., Abdalla D.S. (2013). Cloning and expression of an anti-LDL(-) single-chain variable fragment, and its inhibitory effect on experimental atherosclerosis. mAbs.

[22] Faulin T.D.E.S., Guilherme D.F., Silva A.S., Abdalla D.S.P., Hering V.R., Politi M.J., Maranhão A.Q. (2014). GFP-SCFV: Expression and possible applications as a tool for experimental investigations of atherosclerosis. Biotechnol. Prog..

[23] Cavalcante M.F., Kazuma S.M., Bender E.A., Adorne M.D., Ullian M., Veras M.M., Saldiva P.H.N., Maranhão A.Q., Guterres S.S., Pohlmann A.R. (2016). A nanoformulation containing a scFv reactive to electronegative LDL inhibits atherosclerosis in LDL receptor knockout mice. Eur. J. Pharm. Biopharm..

[24] Michaloski J.S., Redondo A.R., Magalhães L.S., Cambui C.C., Giordano R.J. (2016). Discovery of pan-VEGF inhibitory peptides directed to the extracellular ligand-binding domains of the VEGF receptors. Sci. Adv..

[25] Beppler J., Ben Mkaddem S., Michaloski J., Honorato R.V., Velasco I.T., De Oliveira P.S.L., Giordano R.J., Monteiro R.C., Da Silva F.P., Oliveira P.S.L. (2016). Negative regulation of bacterial killing and inflammation by two novel CD16 ligands. Eur. J. Immunol..

[26] Oliveira C.L.P. (2011). Investigating Macromolecular Complexes in Solution by Small Angle X-Ray Scattering. Current Trends in X-Ray Crystallography.

[27] Weischenfeldt J., Porse B. (2008). Bone Marrow-Derived Macrophages (BMM): Isolation and Applications.

[28] Bancells C., Canals F., Benítez S., Colomé N., Julve J., Ordóñez-Llanos J., Sánchez-Quesada J.L. (2010). Proteomic analysis of electronegative low-density lipoprotein. J. Lipid Res..

[29] Klausner R.D., Donaldson J.G., Lippincott-Schwartz J. (1992). Brefeldin A: Insights into the control of membrane traffic and organelle structure. J. Cell Biol..

[30] Martinez F.O., Gordon S. (2014). The M1 and M2 paradigm of macrophage activation: Time for reassessment. F1000Prime Rep..

[31] Medbury H.J., James V., Ngo J., Hitos K., Wang Y., Harris D.C., Fletcher J.P. (2013). Differing association of macrophage subsets with atherosclerotic plaque stability. Int. Angiol..

[32] Mc Neill E., Crabtree M.J., Sahgal N., Patel J., Chuaiphichai S., Iqbal A.J., Hale A.B., Greaves D.R., Channon K.M. (2015). Regulation of iNOS function and cellular redox state by macrophage Gch1 reveals specific requirements for tetrahydrobiopterin in NRF2 activation. Free Radic. Boil. Med..

[33] Jablonski K.A., Amici S.A., Webb L.M., Ruiz-Rosado J.D.D., Popovich P.G., Partida-Sánchez S., Guerau-De-Arellano M. (2015). Novel Markers to Delineate Murine M1 and M2 Macrophages. PLoS ONE.

[34] Nathan C. (1992). Nitric oxide as a secretory product of mammalian cells. FASEB J..

[35] Moncada S., Higgs E.A. (1995). Molecular mechanisms and therapeutic strategies related to nitric oxide. FASEB J..

[36] Singh U., Jialal I. (2006). Oxidative stress and atherosclerosis. Pathophysiology.

[37] Luoma J.S., Strålin P., Marklund S.L., Hiltunen T.P., Särkioja T., Ylä-Herttuala S. (1998). Expression of extracellular SOD and iNOS in macrophages and smooth muscle cells in human and rabbit atherosclerotic lesions: Colocalization with epitopes characteristic of oxidized LDL and peroxynitrite-modified proteins. Arter. Thromb. Vasc. Boil..

[38] Garlanda C., Dinarello C.A., Mantovani A. (2013). The interleukin-1 family: Back to the future. Immunity.

[39] McDowell T.L., Symons J.A., Duff G.W. (2005). Human interleukin-1α gene expression is regulated by Sp1 and a transcriptional repressor. Cytokine.

[40] McCarthy D.A., Ranganathan A., Subbaram S., Flaherty N.L., Patel N., Trebak M., Hempel N., Melendez J.A. (2013). Redox-control of the alarmin, Interleukin-1α. Redox Biol..

[41] Niemann-Jönsson A., Söderberg I., Lindholm M.W., Jovinge S., Nilsson J., Fredrikson G.N. (2007). Medial Expression of TNF-α and TNF Receptors Precedes the Development of Atherosclerotic Lesions in Apolipoprotein E/LDL Receptor Double Knockout Mice. Int. J. Biomed. Sci. IJBS.

[42] Hashizume M., Mihara M. (2012). Blockade of IL-6 and TNF-α inhibited oxLDL-induced production of MCP-1 via scavenger receptor induction. Eur. J. Pharmacol..

[43] Zhang S., Kim C.C., Batra S., McKerrow J.H., Loke P. (2010). Delineation of Diverse Macrophage Activation Programs in Response to Intracellular Parasites and Cytokines. PLoS Negl. Trop. Dis..

[44] Witztum J.L., Lichtman A.H. (2014). The Influence of Innate and Adaptive Immune Responses on Atherosclerosis. Annu. Rev. Pathol. Mech. Dis..

[45] Miller Y.I., Tsimikas S. (2013). Oxidation-specific epitopes as targets for biotheranostic applications in humans. Curr. Opin. Lipidol..

[46] Miller Y.I., Choi S.-H., Wiesner P., Fang L., Harkewicz R., Hartvigsen K., Boullier A., Gonen A., Diehl C.J., Que X. (2011). Oxidation-Specific Epitopes are Danger Associated Molecular Patterns Recognized by Pattern Recognition Receptors of Innate Immunity. Circ. Res..

[47] Amir S., Hartvigsen K., Gonen A., Leibundgut G., Que X., Jensen-Jarolim E., Wagner O., Tsimikas S., Witztum J.L., Binder C.J. (2012). Peptide mimotopes of malondialdehyde epitopes for clinical applications in cardiovascular disease. J. Lipid Res..

[48] Honold L., Nahrendorf M. (2018). Resident and monocyte-derived macrophages in cardiovascular disease. Circ. Res..

[49] Bobryshev Y.V., Ivanova E.A., Chistiakov D.A., Nikiforov N.G., Orekhov A.N. (2016). Macrophages and Their Role in Atherosclerosis: Pathophysiology and Transcriptome Analysis. BioMed Res. Int..

