# Supplementary material for: Proinflammatory Action of a New Electronegative Low-Density Lipoprotein Epitope
Source: Biomolecules. 2019 Aug 20;9(8):386. doi: 10.3390/biom9080386 (PMC6723646; doi:10.3390/biom9080386)
Supplement: Supplementary file 1 [file biomolecules-09-00386-s001.zip › biomolecules-561233-SI.pdf]

Supporting information

"Proinflammatory action of a new electronegative low-density lipoprotein epitope"

Tanize do Espirito Santo Faulin, Soraya Megumi Kazuma, Gustavo Luis Tripodi, Marcela Frota Cavalcante, Felipe Wakasuqui, Cristiano Luis Pinto Oliveira, Maximilia Frazão de Souza Degenhardt, Jussara Michaloski, Ricardo José Giordano, Daniel Francisco Jacon Ketelhuth, and Dulcineia Saes Parra Abdalla

This file demonstrates additional information about: (A) phage display selection and results data from selection cycles, SDS electrophoresis and Western Blot, demonstrating the specificity of the selected peptides; then, (B) SAXS protocol with explanations about model and parameters used to analyze the data; (C) Macrophage characterization with F4/80 labeling BMDM to confirm macrophage population and positive control with LPS to qPCR experiments.

(A) Phage Display

Biopanning

Phage methods were performed as previously described [1], with modifications. X_6 and CX_8C libraries were screened on immobilized anti-LDL(-) mAbs. A 96-well flat-bottom polystyrene microtiter plate (TPP) was coated with 1 µg/well of purified 1A3H2 or 2C7D5F10 anti-LDL(-) mAb in 50 µL of

sterile PBS pH 7.4, covered with a lid, and incubated overnight at 4°C. After washing twice with PBS, 350 µL of blocking buffer (3% BSA in PBS pH 7.4) were added for 2 h at room temperature (RT). The blocking buffer was removed and 10^9 phages diluted in blocking buffer were added and incubated for 2 h at RT. To avoid binding of phage to Fc of IgG mAbs, 5 µg/mL of mouse IgG was added with the libraries in all rounds. Non-bound phages were removed by washing the wells ten times with PBS. The bound phages were recovered by infection with 200 µL of kanamycin-resistant *Escherichia coli* K91 bacteria at log phase. Infected bacteria were incubated in Luri-Bertani (LB) medium supplemented with 20 µg/mL tetracycline and 10 µg/mL kanamycin, for 18 h at 37°C with vigorous shaking. The supernatant was obtained by centrifugation and phages were precipitated with cold PEG/NaCl (16.7% polyethylene glycol 8,000 in 3.3 M sodium chloride solution) on ice for 2 hours. Precipitated phages were used in the following round. In the second to fourth rounds of biopanning, the procedures were identical to the first round, with an additional step. Infected bacteria were plated in triplicates at serial dilutions onto LB agar medium supplemented with tetracycline, and the number of colonies was counted after an overnight incubation of the plates at 37°C. Selected phage clones that bind to each of the individual mAbs were sequenced to identify the peptide displayed as described [1].

Binding assays

Based on sequence analysis, three phage clones were selected for reactivity with 1A3H2 and seven phage clones were selected for reactivity with 2C7D5F10 following enrichment of the phage display peptide library.

These selected clones were further evaluated for reactivity with their respective mAbs and the non-respective mAb or a mouse IgG was used as a negative control. To this purpose, a 96-well flat-bottom polystyrene microtiter plate was coated with or 50 µL blocking buffer or 1 µg/well of purified 1A3H2 or 2C7D5F10 anti-LDL(-) mAb or mouse IgG in 50 µL of sterile PBS pH 7.4, covered with a lid, and incubated overnight at 4°C. After washing twice with PBS, 350 µL of blocking buffer were added for 2 h at RT. The blocking buffer was removed and 10^8 selected clone phages or fd (wild-type control) diluted in blocking buffer were added and incubated for 2 h at RT. Non-bound phages were removed by washing the wells ten times with PBS. The bound phages were recovered by infection with 200 µL of kanamycin-resistant *Escherichia coli* K91 bacteria at log phase. The number of phage bound to each mAb was determined by serial dilutions onto LB agar medium supplemented with tetracycline and kanamycin, and colony counting after an overnight incubation of the plates at 37°C. To further confirm the specificity of phage interaction, binding assays were also performed in the presence and absence of the corresponding cognate synthetic peptides.

SDS-PAGE and Western Blotting

After expression and purification, peptides-pIII run in an electrophoresis equipment (15% acrylamide gels, 1.5 mm thickness). Samples, with and without β-mercaptoethanol, and the pre-stained protein ladder (Fermentas SM1811) were applied. After running, one of the gels was stained with coomassie blue, the other one was used for protein transfer to nitrocellulose

membrane (Millipore cat nº HATF00010). The transfer tank remained in a 4°C cold chamber and was subjected to 100 volts current for 1 hour. The membranes containing the transferred proteins were incubated with Odyssey blocking buffer (LI-COR Biosciences) under shaking at 4°C overnight. Membranes were incubated with 10 µg/mL of 1A3H2 or 2C7D5F10 MAbs for 2 hours with shaking, at RT. The membranes were washed with 0.05% PBS-Tween pH 7.4 for 25 minutes with buffer changes every 5 minutes and then incubated with IRDye 800CW donkey anti-mouse IgG secondary antibody (LI-COR Biosciences) diluted 1:10,000 for 1 hour under stirring at RT. After washing, membranes were revealed using the Odyssey Fc imager.

ELISA with synthetic peptides

The reactivity of synthetic peptides with anti-LDL(-) mAbs was also confirmed by ELISA. Because the synthetic peptides are very small, they were conjugated to keyhole limpet hemocyanin carrier protein (KLH) to improve their adsorption onto the ELISA plates. Then, a 96-well flat-bottom polystyrene microtiter plate (Nunc Immobilizer, Roskilde, Denmark) was coated with 1500 to 2 ηg/mL of synthetic peptide in 100 mM carbonate buffer (pH 9.6) overnight at 4°C. LDL(-) at 200 ηg/mL was used as positive control. The plate was washed three times with PBS (pH 7.4) containing 0.05% Tween 20 (250 mL/well) and blocked with 2% skimmed milk in PBS for 1h15 min at 37°C. Then, 10 µg/mL anti-LDL(-) mAb diluted in PBS containing 1% skimmed milk were added to the plate, which was incubated for 1.5 h at 37°C. After that, the plate was washed and horseradish peroxidase-conjugated goat anti-mouse IgG was used as a secondary antibody at 37°C for 1 h. The

reactivity of peroxidase was measured using washed plates by incubating with TMB for 10 min at 37°C. The reaction was stopped by adding 0.5 M sulfuric acid, and the absorbance at 450 nm was measured by spectrophotometry using a microplate reader (SynergyTM Mx, Biotek instruments Inc, Winooski, VT, USA).

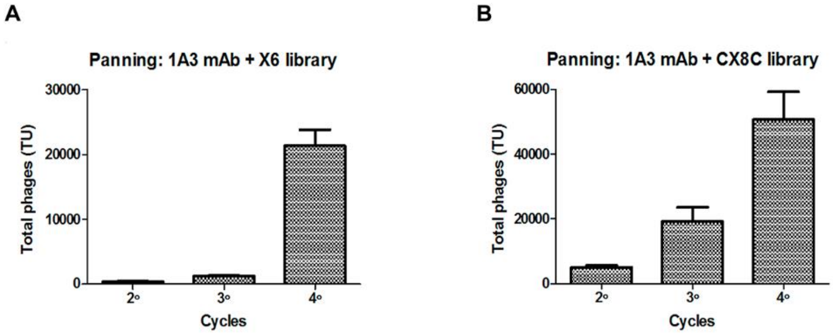

Fig S1. Selection cycles of affinity using phages-displayed X6 and CX8C libraries with 1A3 mAb. Ligand phages were enriched after each selection cycle with 1A3 mAb for the four cycles using X6 (A) and CX8C (B) libraries. The number of transducing units from each cycle is shown in the graphs. The bars indicate standard error of the triplicate mean of the plates.

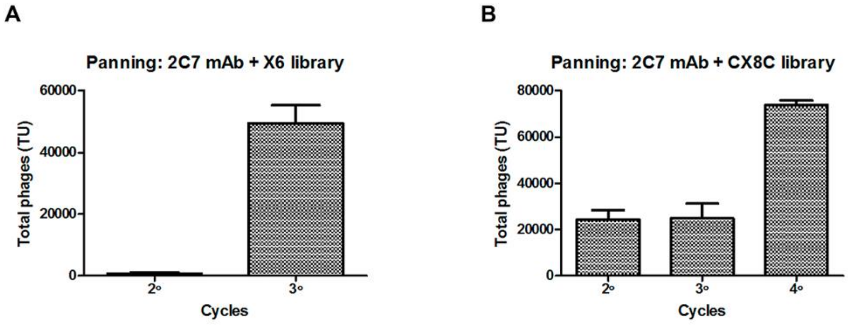

Fig S2. Selection cycles of the phages with X6 and CX8C libraries with 2C7 mAb. Ligand phages were enriched after three selection cycles with 2C7 mAb using X6 (A) and CX8C (B) libraries. There was no need for the fourth cycle with X6 library. The number of

transducing units from each cycle is shown in the graphs. The bars indicate standard error of the triplicate mean of the plates.

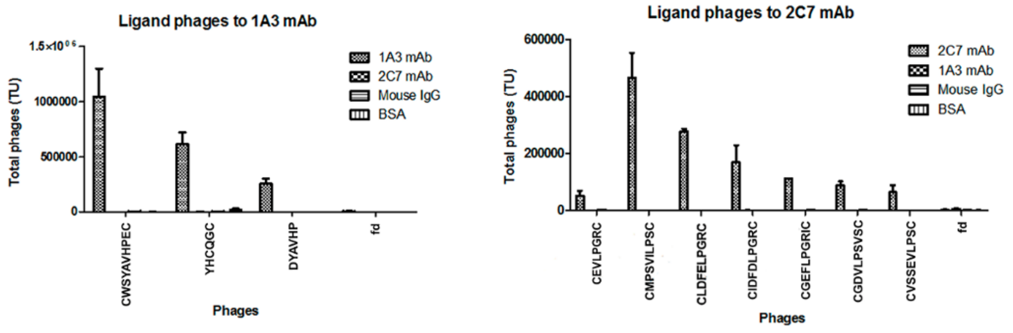

Fig S3. Binding of selected phages to 1A3 (A) and 2C7 (B) mAb immobilized on 96 wells plate. The phages CWSYAVHPEC and YHCQGC presented higher affinity to 1A3 mAb than the phage DYAVHP. Phages CMPSVILPSC and CLDFELPGRC presented higher affinity to 2C7 mAb compared to the other five tested phages. No unspecific binding to 1A3 and 2C7 mAbs, IgG or BSA was observed among the phages. The cutoff used to determine “no binding” was the binding of the Fd phage (wild) to the mABs.

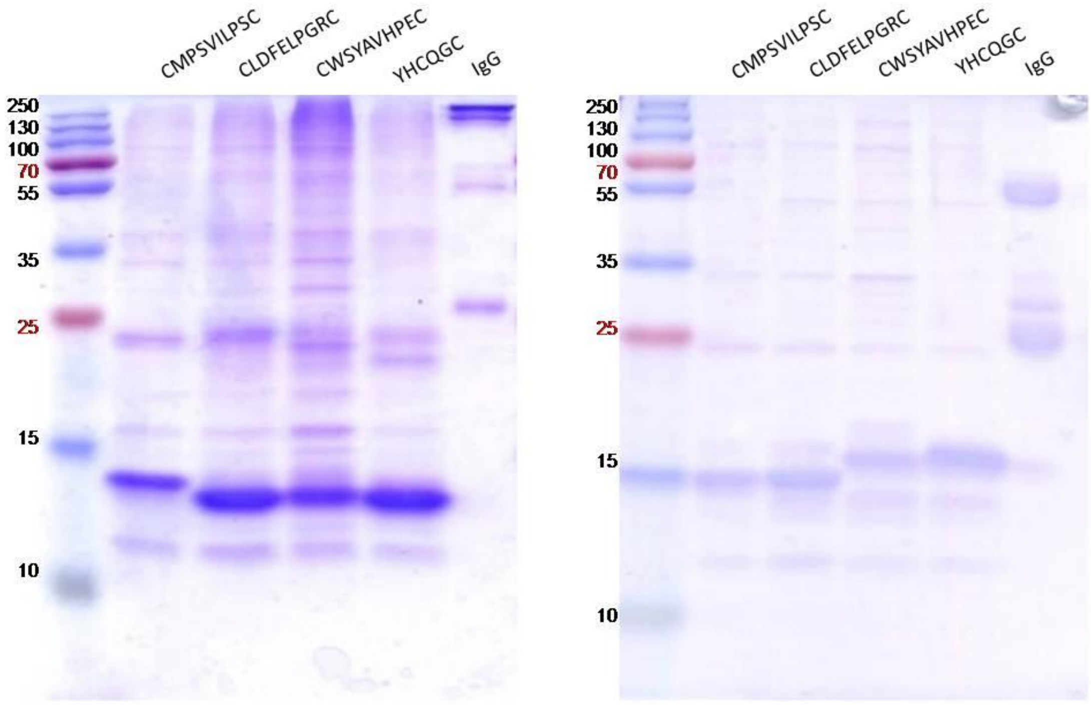

A B

Fig S4. SDS-PAGE electrophoresis of peptide-pIII fusion after purification. The peptides-pIII were purified using nickel resin. Gels in denaturing (A) and non-denaturing (B) conditions show the bands of peptides-pIII with approximately 15kDa.

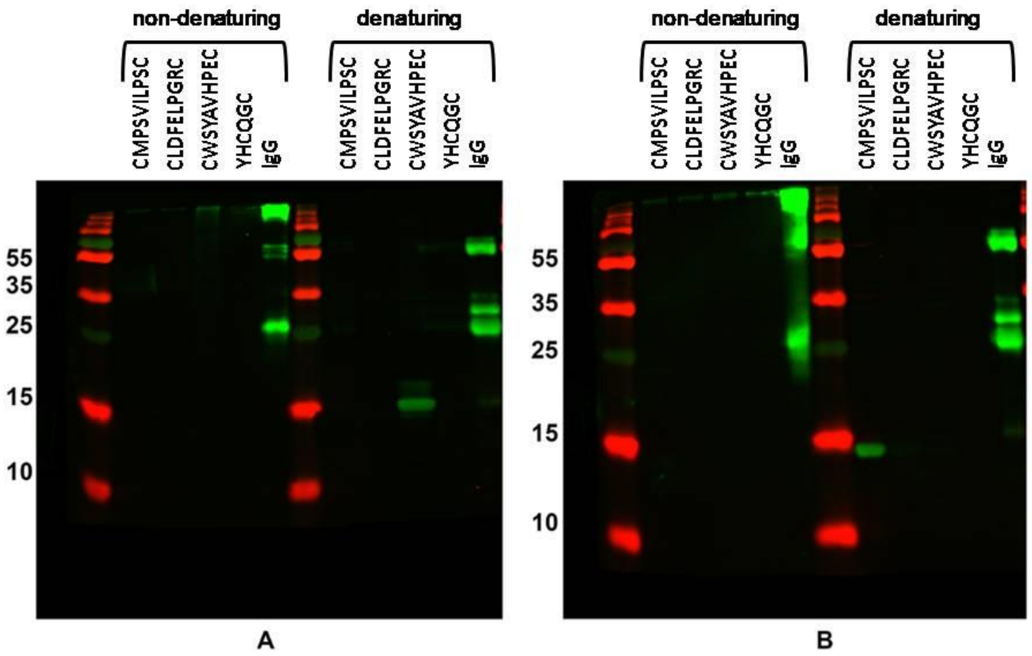

Fig S5. Western Blotting with peptides-pIII. Membrane incubated with 1A3 mAb confirm the affinity of P1-pIII to the antibody (A). Membrane incubated with 2C7 mAb also confirm the affinity of the P2-pIII to the antibody (B). As positive control mouse IgG was used. Red bands: standard of molecular mass.

(B) SAXS structure characterization

SAXS experiments were performed on la Laboratory based SAXS equipment Xenocs-Xeuss^{TM} equipment located at the institute of Physics, University of São Paulo. Samples (2 mg/mL P1A3 or P2C7) were measured in reusable quartz capillaries, which are placed in stainless steel cases. Therefore the holders can be rinsed and reused in order to ensure an optimum background subtraction. The high flux of the instrument, low parasitic scattering and the use of these reusable sample holders was very important due to the very weak scattering of the samples. Two different sample-to-detector distances

were used: 100 and 38 cm. The samples were measured in several 1800s frames. The measurements performed at longer distances were done in two temperatures, 20°C and 36°C. For the short distance the data was collected at 20°C. The collected frames in each case were compared and averaged in order to check sample stability and improve the data quality. The scattering intensities are shown as a function of reciprocal space momentum transfer modulus $q=4\pi\sin(\theta)/\lambda$, where λ is the wavelength of the incident radiation and 2θ the scattering angle. Two modeling approaches were used for describing the experimental data. At first the Indirect Fourier Transformation (IFT) was applied for the data analysis. This approach assumes that the system is composed by identical particles (monodisperse system) and calculates the pair distances distribution function, p(r) [2]. From the shape of the p(r) function it is possible to have indications about the overall shape of the scattering particle and also aggregated states, average radius of gyration R_G and particle maximum size D_{max} [3]. As a second modeling approach it is assumed that the particle can be described as a circular ring of aminoacids. This ring is built by a circular arrangement of spheres and the intensity is calculated by the use of the Debye Formula [4]:

$$I_{\mathrm{mod}}(q) = \sum_{i,j} f_i(q)\, f_j(q) \frac{\sin(qr_{ij})}{qr_{ij}} \qquad (1)$$

where r_{ij} is the distance between the centers of spheres *i* and *j* respectively and f(q) is the form factor of an homogeneous sphere of radius *K*:

$$f(q) = 3\frac{\sin qK - qK\cos qK}{q^3K^3} \qquad (2)$$

The spheres are placed to form a circular ring. Each sphere in this model represents an aminoacid. The only fitting parameter is the ring radius (*R*). The

distance *d* between aminoacids is fixed (3.6Å) and the number *N* of aminoacids in the ring is simply $N = 2\pi R/3.6$. In some cases, it was observed the presence of aggregates in the system. In order to combine the circular ring form factor and the presence of aggregates it was used a structure factor for a globular aggregate with radius of gyration R_G, $S(q) = 1 + S_{c_2} e^{-\frac{q^2 R_G^2}{3}}$. The final model is given by,

$$I_{theo}(q) = Sc_1 I_{mod}(q,R)\left(1 + S_{c_2} e^{-\frac{q^2 R_G^2}{3}}\right) + back \qquad (3)$$

Therefore in this model we have as fitting parameters the overall scale factor Sc_1, the ring radius *R*, the scale factor of the aggregate Sc_2, the aggregate radius of gyration R_G and a constant background *back*.

Table 1. Parameters obtained from the IFT fitting.

Distance	Parameters	Samples	
		**P1A3**	**P2C7**
**100cm**	**R_G [Å] (20ºC)**	53(1)	112(2)
	**R_G [Å] (36ºC)**	67(5)	130(5)
	**D_{max} [Å] (20ºC)**	~120	~200
	**D_{max} [Å] (36ºC)**	~345	~370
	**MW* [kDa] (20ºC)**	~600	~3000
	**MW* [kDa] (36ºC)**	~1000	~9000
**38cm (20ºC)**	**R_G [Å]**	---	6.8(2)
	**D_{max} [Å]**	---	~14.5
	**MW [kDa]**	---	1.54(16)

R_G, radius of gyration; D_{max}, particle maximum size; MW, molecular weight.

Table 2. Parameters obtained from the model fitting assuming circular ring.

Distance	Parameters	Samples			
		P1A3 20ºC	P2C7 20ºC	P1A3 36ºC	P2C7 36ºC
100cm	Sc1	0.0005(1)	0.0004(1)	0.00008(1)	0.0004(1)
	R [Å]	5.7*	5.7*	5.7*	5.7*
	N**	10	10	10	10
	Sc2	9(5)	138(20)	30(15)	137(31)
	R_G [Å]	86(28)	98(4)	71(25)	98(5)
38cm	Sc1	---	0.0006(1)	---	---
	R [Å]	---	5.8(1)	---	---
	N**	---	10.1(2)	---	---

Sc1: overall scale factor; R [A]: Radius; N, Number of amino acids; Sc2: scale factor of the aggregate

*kept fixed.**calculated by using $N=2\pi R/3.6$

(C) Macrophage characterization

Characterization of BMDM by flow Cytometry

After 1 week of differentiation, 1 x 10^6 cells were incubated with F4/80-PE-Cy5 antibody 1:1000 (eBioscience, SanDiego, CA, USA), which is a specific antibody marker for murine macrophages, for 30 minutes in the dark. After incubation, the labeled cells were centrifuged at 400xg, 15 min, at 4 °C. The BMDMs were resuspended in 300 μL of PBS and analyzed in a flow

cytometer (FACSCanto, BD Biosciences, San Diego, CA, USA) (Fig.6A). The cells were considered as differentiated when the labeling was greater than 95%. We compared RAW 264.7, a classical macrophage cell line with BMDM. Both cells have no difference in F4/80-Cy5 labeling

a

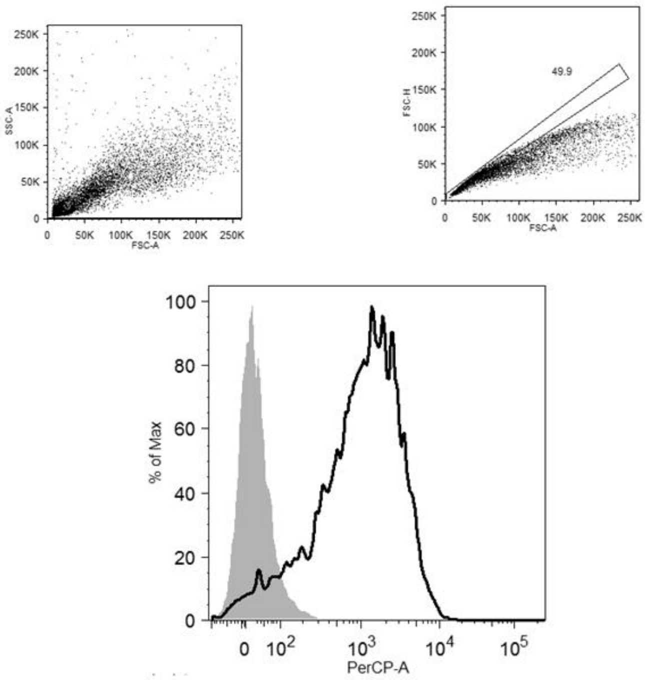

b

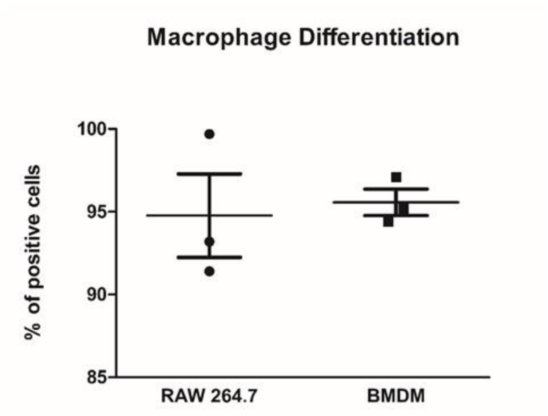

Fig S6. Macrophage Differentiation. To confirm that bone-marrow derived cells were

differentiated into macrophages, BMDMs were labeled with F4/80-PE-Cy5 and analyzed by flow cytometry (a). The RAW 264.7, a murine macrophage cell line was used as the positive control (b).

BMDM activation by LPS

As a positive control for qPCR experiments, BMDMs (10^5 cells/well) were incubated with 10 μg/mL LPS or 100 μg/mL LDL (-). Total RNA was extracted with TRIzol (Life Technologies, Carlsbad, CA, USA) following manufacturer instructions. The extracted RNA was quantified using spectrophotometry. cDNA was constructed from RNA samples by RT-PCR with Superscript Vilo (Life Technologies, Carlsbad, CA, USA). qPCR was performed using the 7500 Fast Real-Time PCR System (Applied Biosystems, Carlsbad, CA, USA) to analyze NOS2, COX-2, TNF-α, IL-1α, TGF-β and IL-10 mRNA expression (Fig S7). The sequence of primers was described in the table 3.

Table 3. Primers sequence

Gene	Forward (5'-3')	Reverse (5'-3')
Cox2	TGGTGCCTGGTCTGATGATG	GTGGTAACCGCTCAGGTGTTG
Tnfa	GGTGCCTATGTCTCAGCCTC	CACTTGGTGGTTTGCTACGA
Il10	GTACAGCCGGGAAGACAATAA	GCATTAAGGAGTCGGTTAGCA
Nos2	CAGCTGGGCTGTACAAACCTT	CATTGGAAGTGAAGCGGTTCG
Il1a	AGTATCAGCAACGTCAAGCAA	TGATCTGGGTTGGATGGTC
Tgfb	ACGGAATACAGGGCTTTCG	GTTCATGTCATGGATGGTGC
Gadh	TCACTCACGGCAAATTCAACG	TAGACTCCACGACATACTCAGC

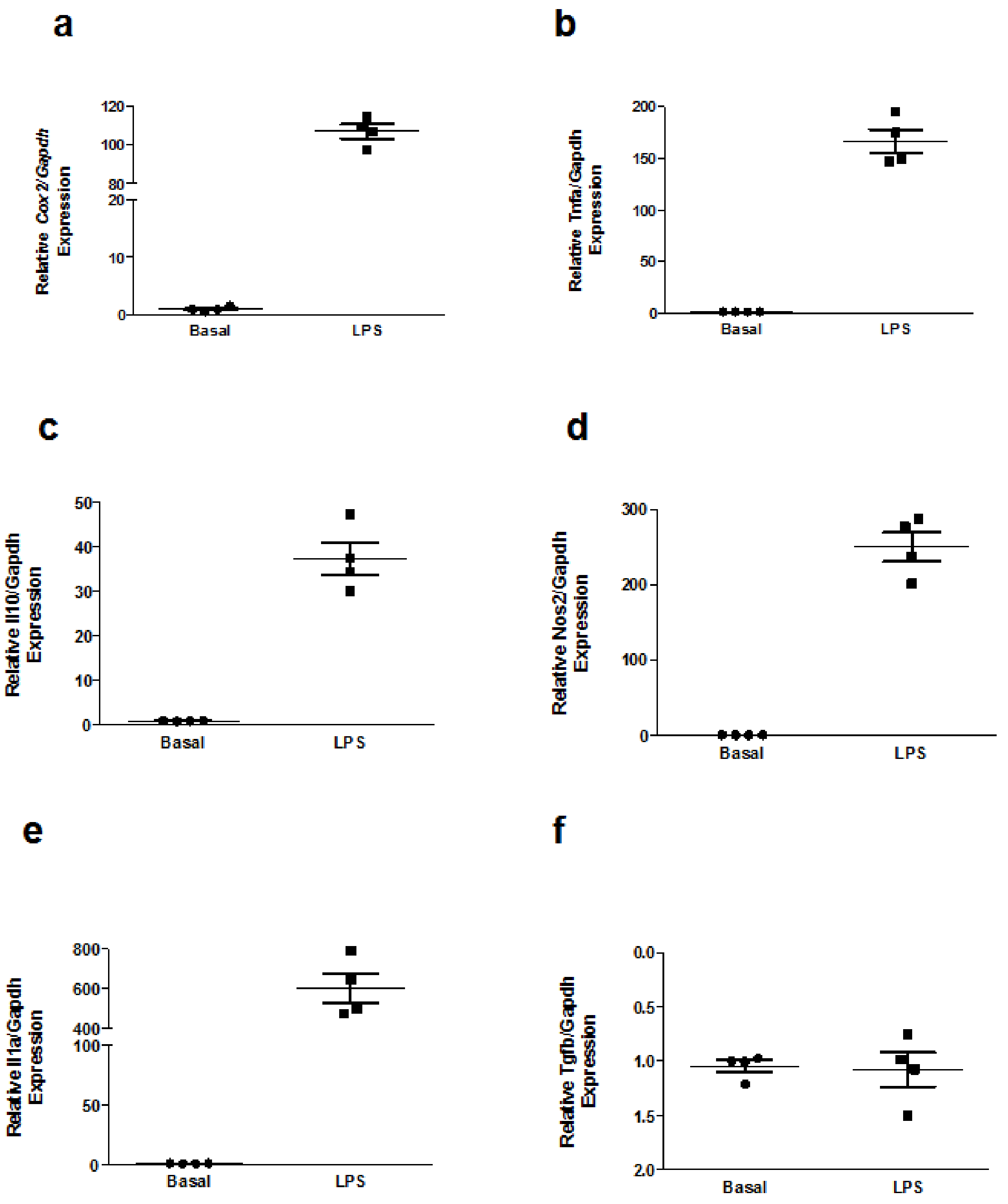

Figure S7. Effect of LPS on mRNA expression in bone marrow-derived macrophages evaluated by qPCR. The mRNA of the proinflammatory mediators COX-2, TNF-α, IL-1α, and NOS2 (a, b, e and d, respectively) as well as the anti-inflammatory cytokines IL-10 and TGF-β mRNA (c and f) were evaluated by qPCR.

Evaluation of BMDM polarization by flow cytometry:

For this assay, 10^6 cells/well were incubated for 48 hours with 100 µg/mL P2C7 or PBS as the negative control. After incubation, macrophages were washed with PBS and removed from the plate with cold PBS (plus 10 mM EDTA). The cells were stained with anti CD206 - FITC (Invitrogen, MR5D3) and anti-F4/80 – PE – Cy5 (eBioscience, BM8).

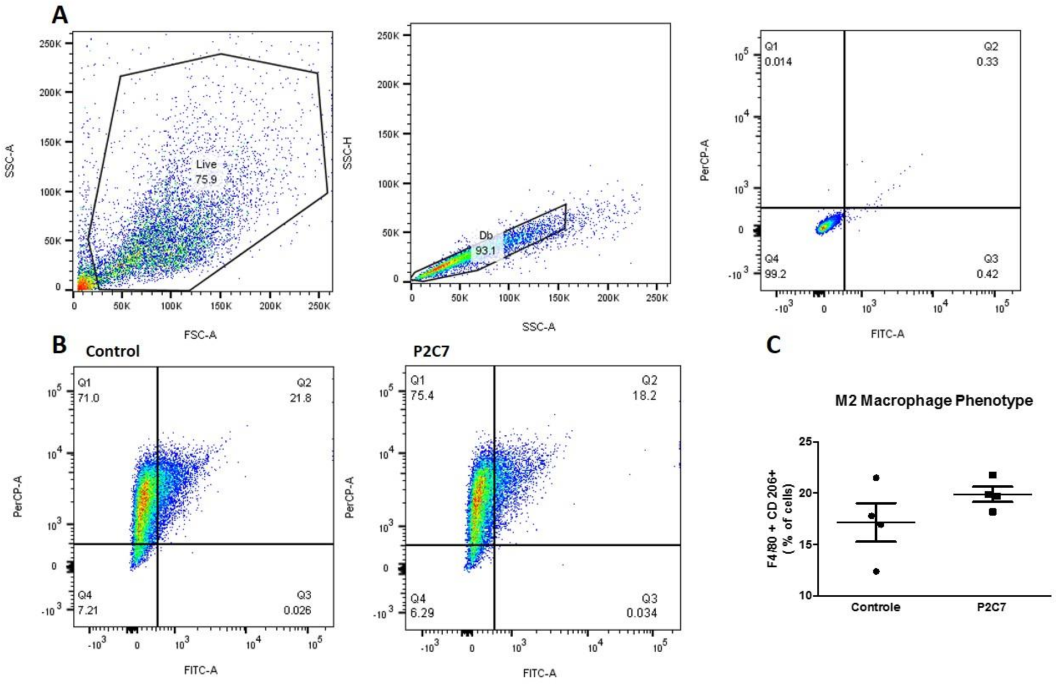

Fig S8. M2 macrophage phenotype. 1 x 10^6 BMDMs were treated with 100 µg/mL of P2C7 or non-treated as negative control for 48 hours. After the incubation, the cells were washed and stained with anti-F4/80-PE-Cy5 and anti-CD206-FITC. After selecting the gates with non-stained cells (A), the M2 phenotype (F4/80+CD206+) was accessed by flow cytometry (B and C). The statistical analyses were performed with T-test, (n=4).

References

[1] J.S. Michaloski, A.R. Redondo, L.S. Magalhães, C.C. Cambui, R.J. Giordano, Discovery of pan-VEGF inhibitory peptides directed to the extracellular ligand-binding domains of the VEGF receptors, Sci. Adv. 2 (2016) e1600611. doi:10.1126/sciadv.1600611.

[2] O. Glatter, A new method for the evaluation of small-angle scattering data, J. Appl. Crystallogr. 10 (1977) 415–421. doi:10.1107/S0021889877013879.

[3] C.L. Pinto Oliveira, Investigating Macromolecular Complexes in Solution by Small Angle X-Ray Scattering, in: Curr. Trends X-Ray Crystallogr., InTech, 2011: pp. 15–24. doi:10.5772/30730.

[4] P. Debye, Zerstreuung von Röntgenstrahlen, Ann. Phys. 351 (1915) 809–823. doi:10.1002/andp.19153510606.
